# A Review of Infectious Diseases Associated with Religious and Nonreligious Rituals

**DOI:** 10.1155/2021/1823957

**Published:** 2021-12-06

**Authors:** Kiran Gajurel, Stan Deresinski

**Affiliations:** ^1^Division of Infectious Diseases, Carolinas Medical Center, Atrium Health, Charlotte, NC, USA; ^2^Division of Infectious Diseases and Geographic Medicine, Stanford University School of Medicine, Stanford, CA, USA

## Abstract

Rituals are an integral part of human life but a wide range of rituals (both religious and non-religious), from self-flagellation to blood brotherhood to ritual sprinkling of holy water, have been associated with transmission of infections. These infections include angiostrongyliasis, anthrax, brucellosis, cholera, COVID-19, cutaneous larva migrans, Ebola, hepatitis viruses, herpes simplex virus, HIV, human T-cell leukemia virus (HTLV), kuru, *Mycobacterium bovis*, *Naegleria fowleri* meningoencephalitis, orf, rift valley fever, and sporotrichosis. Education and community engagement are important cornerstones in mitigating infectious risks associated with rituals.

## 1. Introduction

Rituals are part of the fabric of human society. They are deeply intertwined with traditional cultures and may provide psychological benefits and have an adaptive function in society, facilitating cooperation among individuals [1]. Some rituals, however, have been associated with the acquisition of infectious diseases. In this review, we explore both religious and non-religious rituals associated with infection using search terms “rituals,” “religious rituals,” “rites,” and “religious rites” combined with “infection” and “infectious disease” in the PubMed database limited to the English language through September 2021. Relevant references from the retrieved articles were also reviewed for additional information. A wide variety of infections ranging from virus to prion was found to be associated with rituals and is summarized in Table 1.

## 2. Rituals and Viral Infections

### 2.1. Self-Flagellation and Human T-Cell Leukemia Virus (HTLV) and Hepatitis C Virus

Self-flagellation is a religious practice carried out by Shia Muslims commemorating the martyrdom of Husayn ibn Ali. It was also practiced in Middle Age Christian life, and, nowadays it is still used by some groups such as Opus Dei communities. In this ritual, the body is whipped with sharp instruments causing bleeding. Tang et al. recently described asymptomatic HTLV-1 infection thought to be acquired via self-flagellation among 10 heterosexual Muslim men, mostly of Indian and Pakistani origin, living in the United Kingdom [2]. The infection was discovered during screening for blood donation (including cord blood) or in vitro fertilization. There were no other concomitant blood borne pathogens identified except in one patient who was found to have hepatitis C virus co-infection. Eight of 10 men had a history of sharing equipment for self-flagellation. Five couples were serodiscordant and eight had single lifetime sexual partners. There were no obvious risk factors for HTLV-1 acquisition although mother to child transmission could not be excluded in eight of them. The mother of one patient was HTLV-1 seronegative, and one other patient had a negative HTLV serology nine years earlier, thus implying HTLV-1 acquisition later in life.

In another study from Australia, 7 (28%) of 25 HLTV-1 positive prospective blood donors between 2012 and 2018 were reported to have engaged in self-flagellation [3]. All seven were adult males born in India and Pakistan. Other risks factors for HTLV-1 were not assessed, but since India and Pakistan are not HTLV endemic areas, self-flagellation was believed to be a mean of acquisition of HTLV-1 in these patients.

Self-flagellation has also been found to be a risk factor for hepatitis C infection, and it has been reported to account for 5% of hepatitis C infections among Iranian blood donors [4].

### 2.2. Orf

Orf, caused by a zoonotic *Parapoxvirus*, has been reported during Eid alAdha due to accidental hand lacerations during animal handling for sacrifice [5–8]. Lamb sacrifice and consumption also occur in other religions and, as a consequence of global migration, orf is likely to be encountered in places where it had never been present before[9]. Orf can mimic other cutaneous infections like anthrax, tularemia, and mycobacterial infections, and thus familiarity with this infection and its association with religious festivals will avoid unnecessary treatment and investigation.

### 2.3. Blood Brotherhood and Infectious Diseases

Blood brotherhood rituals involve superficial cutting of fingers, hands, or forearms and pressing the bleeding areas together as a sign of bonding and loyalty. This ritual has been practiced for centuries all over the world and remains prevalent to this day. Unfortunately, this ritual carries a risk of transmission of blood borne pathogens, but studies supporting disease transmission are limited [10, 11].

In 2007, a study in Turkey revealed that almost one-fourth of 2311 high school students (both males and females) had participated in blood brotherhood rituals, and nearly one-third of 1615 (most of whom had already participated in this ritual before) intended to participate in such rituals in the future [12]. About 29% of blood brotherhood participants had a history of hepatitis B infection, but further details regarding the activity of hepatitis B infection and disease transmission were not provided [12].

In one study, all 7 (13%) of 52 blood donors who were found to have antibody to hepatitis C virus admitted having blood brothers or sisters while in school [13]. None of them were reported to have other known risks for acquiring hepatitis C.

Another study reports a case of possible HIV transmission in an adult through blood brotherhood ritual [14]. The same paper also described a case of acute symptomatic hepatitis B infection in a 17-year-old male, 65 days after participating in blood brotherhood rituals [14]. This patient tested positive for hepatitis B surface antigen, core IgM, and e antigen. Six months later, surface antigen turned negative, but core total IgG and surface antibody converted positive. One other person also had a similar icteric illness. One of the blood brothers was subsequently found to be a hepatitis B carrier. However, without genomic sequencing true transmission events cannot be proven.

### 2.4. Other Rituals Involving Blood Including Scarification and Female Genital Mutilation

The cultural practice of bloodletting was common in the 1980s in parts of rural Africa. This, along with group circumcision and scarification in certain African cultures performed with the use of shared instruments, might have contributed to the spread of HIV [15]. Similar cultural practices (including blood suction in folk medicine) in other parts of the world may have been responsible for the spread of hepatitis B and C viruses [11, 16, 17]. An earlier study found a statistically higher prevalence of hepatitis B surface antigen among South African Bantu community who underwent ritual scarification by witch doctors suggesting transmission of hepatitis B via unsterilized instruments [18]. One study described possible HIV transmission in a young woman in rural Africa during a ceremony which involved cutting the skin of the traditional healer followed by the subject with the same instrument [19].

Ritualistic female genital mutilation, including Gishiri cutting (a form of female genital mutilation practiced in parts of Africa), performed for a variety of purposes such as the transition of girls into womanhood, has been associated with not only viral infections like HIV, herpes simplex virus (HSV), and hepatitis B, but also with *Clostridium tetani, Chlamydia trachomatis, Neisseria gonorrhoeae,* syphili*s,* and *Trichomonas vaginalis* [20, 21].

### 2.5. Widow Inheritance and HIV and Other Similar Rituals

In some African cultures, widows and widowers are considered “impure” and undergo ritual purification to prevent ill fortune. While the widowers are cleansed with water and herbs, the widows undergo “pita-kufa” or “kutchinga” which involve unprotected sexual intercourses with the “purifiers,” often the deceased's relatives, who then “inherit” the widows [22, 23]. The sexual rituals are also observed during other special occasions like harvesting, birth, marriage, and death of relatives. Unfortunately, the sexual cleansing ritual is associated with increased risk of contracting HIV [22]. The risk appears to be even higher when nonrelatives (compared to relatives) inherit the widows for sexual rites [22]. These rituals are currently unpopular, with one study showing only 12% people surveyed in Mozambique wanting to continue the tradition [23].

In certain African communities. ceremonial rites of adolescent girls around the time of puberty include rituals like kusasafumbi (cleansing the dust), kuchotsa (removing dust), and kutaya mafuta (spilling the oil) where the girls are encouraged to have sexual activity with boys [24]. Similarly “disco funerals” (which involves music and dance often to raise money for funeral services) are often venues for meeting places of young girls and boys and the music and dance intermingled with alcohol and drugs often result in unprotected and sometimes forced sexual activities in communities with high HIV prevalence[25]. In rural Africa, several other cultural practices are observed including cokoloa (wife inheritance), mitala (polygamy), cimeta tsitsi (deceased wife replacement), and hyena ritual (where girls are expected to demonstrate that they have grown up by having sexual activity with a group of boys) [26, 27]. All these rituals can potentially increase HIV transmission in the community.

### 2.6. Rift Valley Fever (RVF)

Outbreaks of RVF have occurred primarily in Africa among those whose livelihood depends on livestock. In addition, ritual sacrifice of ram during Eid al-Adha festival can lead to RVF outbreaks (via aerosolization and contact with body fluids/tissue) [28, 29]. Importation and smuggling of animals from Somalia for sacrifice during Eid al-Adha have also led to outbreaks of RVF in nonendemic countries [30]. Ritualistic slaughtering of animals also occurs during wedding ceremonies and dowry payments with a potential to cause an outbreak [29]. During the 2006–2007 RVF outbreak in Kenya, community prayer meetings held by religious leaders involved animal slaughtering, and this could have paradoxically exacerbated the outbreak [29].

### 2.7. Ebola

Ebola virus can be transmitted via direct contact through nonintact skin or mucous membrane with body fluids and fomites. In addition, traditional mortuary rituals that facilitate contact with deceased's body fluids have been identified as risk factors for Ebola transmission [31]. Ritual washing, cleaning and clothing the deceased body in preparation for burial, hand washing in a common bowl, and then touching the deceased body as sign of “love touch” by the relatives are common practices in certain African communities that have been shown to propagate the spread of Ebola virus [32–34]. The presence of higher viral load among the deceased ones (compared to survivors) and prolonged contact with the deceased body during burial rituals can help in amplifying secondary cases [34, 35]. In a study conducted during the 2013–2016 Ebola outbreak in West Africa, 83 (52%) of 159 people who had contact with the Ebola victim after death fell ill (although Ebola infection was not confirmed in all of them) [36]. In another report Ebola virus seropositivity was found to be higher among people who participated in burial rituals compared to those who did not [37].

Laying over the deceased body of prominent traditional healers is another means of Ebola spread in the community [31]. There are also reports of traditional healers cutting the skin of Ebola victims and rubbing herbal medicine in the cut wound and subsequently getting infected themselves and spreading the infection to others through this practice [31]. Healing prayer sessions (involving laying hands on the sick) have also contributed to Ebola transmission [31].

### 2.8. Neonatal HSV Infection

Jewish male infants undergo ritualistic circumcision, called bris (brit) milah. The procedure involves “metzitzah”, where after circumcision the mohel (ritual circumciser) orally suctions blood from the penis. This process can lead to HSV transmission from the mohel (who could be asymptomatically shedding HSV in the mouth) to the infant [38]. A 2014 review found 30 cases of neonatal HSV1 infection (with 2 deaths) following Jewish ritual circumcision [39]. The true burden of neonatal HSV infection associated with this ritual is likely underestimated due to under reporting [40].

### 2.9. Religious Gatherings and COVID-19

Religious gatherings have been venues of COVID-19 outbreaks. A massive outbreak of COVID-19 in the city of Daegu in South Korea early in the pandemic was linked to a religious sect affiliated to Shincheonji Church of Jesus [41, 42]. The infection was amplified by the worshippers participating in various rituals including praying and singing hymns in crowded settings without wearing face masks [41]. In one study, 35 (38%) of 92 church attendees developed laboratory confirmed COVID-19, with 3 deaths and an additional 26 cases linked to church attendees occurring in the community[43]. In another study, 52 of 61 people who attended a choir practice contracted COVID-19 (30 confirmed and 20 probable cases) linked to the index superspreader within the group [44]. There are also several other reports of pilgrims acquiring COVID-19 during mass gatherings and subsequently spreading the infection on their return to their homeland [45–52].

## 3. Rituals and Bacterial Infection

### 3.1. Ritual Sprinkling of Holy Water

There have been several reports of infection associated with ritual sprinkling of holy water [52–55]. In one instance an adult male patient with tibial fracture required a rectus abdominis muscle flap with split skin graft [56]. Unfortunately, the donor graft site developed recurrent *Klebsiella* infection. It was eventually discovered that the patient was sprinkling holy water on the surgical site. Culture of the holy water contained in the bottle grew the same *Klebsiella* species.

In another report, an 11-year-old boy with recalcitrant epilepsy necessitating mechanical ventilation was found to have recurrent multi drug resistant (MDR) *Acinetobacter baumannii* pneumonia [57]. This bacterium was also cultured from the urine and skin area near the percutaneous gastrojejunal tube. The antibiogram of this *A. baumannii* was identical to the one isolated from his prior respiratory culture. Further investigation revealed that the patient's mother had been regularly sprinkling holy water contained in a plastic bottle for several months on the patient's skin, mouth, and the feeding tube area. Interestingly, the parents had large stores of holy water obtained from seven churches in Moscow and a Jordanian river, and MDR *A. baumannii* was recovered in culture from both.

Another report described an adult male with *Pseudomonas aeruginosa* pneumonia while recuperating from multiple injuries [58]. His aunt was observed sprinkling holy water on the patient and culture of water from the bottle grew *P. aeruginosa* that was indistinguishable on phage typing from that isolated from the patient. There were no similar isolates identified in the hospital. While whole genome sequencing was not performed in these reports, the circumstantial evidence supported ritual sprinkling of holy water as the source of infection.

In many cultures and faiths, holy water is collected from rivers and religious shrines, and ritualistic sprinkling of holy water is believed to cure ailments. Unfortunately, bacterial contamination of water sources in religious shrines is not uncommon. And furthermore contamination due to “unholy” pathogens already present in collecting vessels can occur [59]. In one study, holy water fonts from churches in Spain were found to be alarmingly contaminated with bacterial pathogens [60]. In a study of 76 water sample from Buddhist temples in Thailand, only 12% water samples met WHO standards for potability [61]. In another study, water collected from 2 religious shrines in Europe and from a Jordanian river was found to be contaminated with *Candida* species and various bacteria including *P. aeruginosa*, Stenotrophomonas, *Aeromonas hydrophila,* and *A. baumannii* [54]. It is, however, possible that some of these pathogens may have been introduced later during transport and storage [59, 62].

Holy water fonts in religious sites have been found to be contaminated with dirt, molds, worms, and their eggs [63]. One report mentioned a person developing rash on the forehead after blessing herself with holy water from a church water font that was found to be contaminated with worms [63]. Moreover, injection drug users have been found cleansing their syringes in church water fonts [63].

### 3.2. *Mycobacterium bovis*

A recent study described acquisition of *M. bovis* infection in a 43-year-old Tunisian immigrant living in France [64]. On her recent visit to native Tunisia, she participated in Aid-el-Kebir (Muslim festival of sacrifice that honors the willingness of Abraham to sacrifice his son, Ismael, in response to God's command) in which she washed the viscera (including the lungs) of a “veterinary uncontrolled” slaughtered sheep for two hours in an enclosed space at home before cooking and consuming it with her family. There was no injury reported during the process. Twenty-two days later, upon return to France, she started having fever, cough, anorexia, and weight loss. Chest imaging showed a left lower lung abscess and a right lung infiltrate. The diagnosis was delayed, and, about 5 months after the ritual, her sputum was found to be positive for *M tuberculosis* complex on GeneXpert. The patient responded to standard antitubercular therapy. A culture isolate was eventually identified as *M. bovis,* and whole genome sequencing demonstrated its close similarity to a strain found in Algeria (which borders with Tunisia). Although *M. bovis* infection in the slaughtered sheep could not be confirmed because specimens were not available for analysis, the development of symptoms soon after participating in the ritual and genetic relatedness of *M. bovis* to an Algerian strain suggested the infection was acquired (through aerosolization) during cleaning the viscera of the infected sheep during her recent visit to Tunisia.

### 3.3. Brucellosis

Five cases of brucellosis were reported following ceremonial slaughtering of sheep in Ethiopian-born Jews in Israel [65]. None had raised herds or consumed unpasteurized milk products, and the diagnosis was delayed by up to 92 days following symptom onset [65]. The ceremony involves slaughtering, skinning, and evisceration of sheep procedures that can cause aerosolization of infective bacterial particulates leading to pulmonary disease and subsequent dissemination to other organs.

### 3.4. Cutaneous Anthrax

In some folk cultures, there is a tradition of smearing slaughtered animals' blood on the forehead. In one report, two brothers aged 5 and 6 presented with forehead and periorbital swelling with a necrotic center [66]. A week prior to the presentation, their father had smeared the blood of a slaughtered cow on their forehead. The wound cultures of both brothers were confirmed as *Bacillus anthracis*. Both of them recovered with intravenous penicillin therapy. The authors speculate that this traditional ritual explained other reported cases of facial anthrax in children in the region.

### 3.5. Rituals and Cholera

The “Kumbha Mela” is the largest religious gathering on the banks of holy rivers in India where millions of Hindu devotees gather every 12 years. In 2013, an estimated 120 million people attended the festival. Ritualistic bathing and dipping in the holy rivers are a common practice among the devotees during these festivals. Cholera epidemics (due to fecal contamination of the river water from overcrowding and lack of sanitation) dating back to as early as 1817 have occurred during these festivals [67–69]. The 1817 cholera outbreak during the festival may have contributed to the perpetuation of 1817–1824 Asiatic cholera pandemic. Ritualistic drinking of holy water (in addition to inadvertent drinking during bathing rituals) by the pilgrims constitutes an important mechanism of cholera spread [68]. Although no large-scale cholera epidemics have occurred in recent years due to improved public health measures, there remains a risk of an impending outbreak. In 2001, *Vibrio cholerae* was detected in 7 (23%) of 31 fecal samples or rectal swabs during an investigation of increased diarrheal diseases in the festival [70]. A major epidemic was thwarted due to appropriate measures taken by the health authorities.

In the 2013 Kumbha Mela, an increased incidence of other diarrheal diseases and respiratory infection was also observed a few days after bathing rituals[69]. An alarming 130-fold increase in bacterial load in the river water in the 2015 Kumbha Mela suggested significant human contamination [71]. Increase in antimicrobial resistant bacteria including those harboring blaNDM1 is another public health challenge [72]. Besides waterborne diseases, transmission of SARSCoV2 and subsequent spread in the community have also been reported [51, 52].

Cholera outbreaks have also been observed following meals served after funeral events in Africa [73, 74]. Certain communities in Guinea-Bissau observe 3–5 day long funeral events [74]. The deceased ones are washed at night and shrouded with funeral cloth and taken out of the funeral house during the day and again taken back to the funeral house at night until burial. These events are followed by a community meal. During the 1994 cholera epidemic in Guinea-Bissau, cholera occurred more frequently among people who ate at the funeral and touched the deceased body during transporting, washing, and shrouding [74]. The attack rate was less pronounced in villages where the bodies were disinfected after death before the ritualistic bathing and shrouding [74].

## 4. Rituals and Fungal Infection

### 4.1. Sporotrichosis

Extensive body tattooing has been observed in Samoa for centuries. In Samoan tattooing, the teeth of the tattoo comb are made of boar's bone and the pigment is made up of soot and ink-latter obtained by crushing the seeds of candle nut tree. One case report described a 36-year-old Samoan immigrant in Australia who developed culture proven cutaneous sporotrichosis following traditional body tattooing [75].

## 5. Rituals and Parasitic Infection

### 5.1. Angiostrongyliasis

*Angiostrongylus cantonensis*-related eosinophilic meningitis usually occurs following ingestion of vegetables contaminated with its larvae and occasionally after ingesting slugs on a dare [76]. A divine reason for ingesting raw mollusks has also been invoked. Followers of Afro-Brazilian religions (Candomblé and Umbanda) may be required to ingest raw mollusks while performing their religious rituals and sacrificial offerings. A recent study described a cluster of 3 cases of *A. cantonensis*-related eosinophilic meningitis after consuming raw mollusks as a part of religious rituals in this community [77]. One patient with concomitant neurosyphilis presented with headache and was treated with penicillin and albendazole. Two other patients had atypical presentations with extremity pain (one also had testicular pain) in addition to headache and fever and were treated with ivermectin and steroids. All three patients had peripheral eosinophilia and CSF white cell counts ranged from 190 to 720 cell/mm^3^ (with 27–35% eosinophils). The diagnosis was serologically confirmed (serum positive ELISA and Western blot). All three patients improved.

### 5.2. Cutaneous Larva Migrans

Cutaneous larva migrans associated with side rolls, known as “angapradakshinam” (Sanskrit: Anga-body part; pradakshinam-revolution around a temple) has been reviewed previously [38, 78, 79]. In this ritual, devotees lay on the ground and roll sideways in the temple premises. This maneuver brings the bare part of the body in contact with the temple floor infested with hookworm larvae from stray dogs.

### 5.3. *Naegleria* Meningoencephalitis

*Naegleria fowleri* related meningoencephalitis is associated with ritual ablution (involving nasal irrigation) and has been reviewed before [38].

## 6. Rituals and Prions

Kuru, now an extinct disease, was endemic in Papua New Guinea for much of the twentieth century. This neurodegenerative disease caused by prions is characterized by ataxia and emotional alterations and wasting leading invariably to death within two years from symptom onset and has a long incubation period of up to more than 50 years [80–82]. The disease was related to ritualistic transumption in which the decedents' bodies (including the infected brain) were consumed by mourners. With the prohibition of ritualistic transumption in the 1950s, the number of deaths from Kuru declined sharply, with last Kuru related death reported in 2005 [82, 83].

## 7. Other Rituals and Infectious Diseases

Infections associated with mass gatherings (including Haj pilgrimage) have been previously reviewed [84]. Wedding ceremonies have been venues of COVID-19 spread [85, 86]. Holy Communion, a Christian tradition of sharing bread and wine from a common chalice (sometimes using a common spoon) carries a theoretical risk of infection transmission through saliva (including SARSCoV2), but there has never been a reported proven transmission event [87, 88].

There are many other rituals which carry the risk of infection transmission, but evidence based studies are lacking. The association between schistosomiasis and Baptism (a Christian tradition where the body is immersed in water) needs further studies [89]. Rituals like crucifixion among the Roman Catholics, Thaipusam ceremony in Malaysia which involves tongue and cheek piercing, and Bisket Jatra (tongue piercing festival) in Nepal to commemorate the local New Year carry risk of transmission of blood borne pathogens [90, 91]. In some African cultures, saliva is thought to possess special powers and is used in various rituals including the treatment of wounds and snake and scorpion bites [92]. In some cultures, traditional healers spit on the patients and suck their body parts, and the newborn child is blessed by elders by spitting on the face. Similarly, initiation rituals of young girls involve passing off ceremonial beads (contaminated with saliva) using mouth. Rituals like these have a potential to transmit various infections including HHV8 [92].

## 8. Discussion

A wide range of infections ranging from virus to prions are associated with various religious and non-religious rituals. Education is crucial in mitigating the risk of acquiring these infections. Educational intervention decreased the intention to participate in future blood brotherhoods from 30 to 21% in a study of high school students[12]. Similarly, deworming of stray dogs with mebendazole around the time of Nallur temple festival in Sri Lanka significantly reduced the incidence of cutaneous larva migrans among devotees who performed side rolls [93].

Modification of cultural practices (e.g., using cleansing herbs rather than actual sexual acts during initiation rites of young girls) can help reduce transmission of HIV[27]. It should be noted though that traditional rituals are deeply ingrained in the human society, and changes in local customs and cultures should be dealt with tactfully. As an example, implementation of safe burial practice is a cornerstone in preventing community spread of Ebola virus, but disposing of bodies in unmarked bags and rapid burial in unmarked graves have caused mistrust among the locals leading to secret burials and violence on health care workers[36, 94, 95]. Enforcing radical changes in burial practice during plague outbreak in the early twentieth century in Madagascar was also met with mistrust and confrontation with the colonial authorities which led to hiding of the sick and the dead relatives [96]. On the other hand, education and community engagement have been demonstrated to change behavioral practice including safe and dignified burial of Ebola victims [97]. Similar community level cooperation including bans in Northern Kenya on animal slaughter around the time of Eid al-Adha was instrumental in decreasing RVF mortality in Kenya [98].

## 9. Conclusion

Infections associated with rituals are a relatively unexplored territory. Most studies of infections associated with rituals are limited to small case series or reports. While most reports lack conclusive evidence such as a demonstration of genomic identity of pathogens, the relationship to ritual activities is clear in most cases. Further studies are needed to assess the disease burden and the development of approaches to mitigation of the risk of infection transmission.

## Figures and Tables

**Table 1:** Rituals and infectious diseases.

Rituals	Associated infection
Self-flagellation	HTLV-1 and hepatitis C
Ritualistic consumption raw mollusks	*Angiostrongylus cantonensis*-related eosinophilic meningitis
Ritualistic animal sacrifice	*Mycobacterium bovis*, orf, brucellosis, cutaneous anthrax, and rift valley fever
Sprinkling of holy water	Bacterial infections (surgical site, pneumonia)
Blood brotherhood and other rituals involving blood (e.g., group circumcision, skin scarification, bloodletting, and female genital mutilation)	HIV, hepatitis B, hepatitis C, *Clostridium tetani, Chlamydia trachomatis, Neisseria gonorrhoeae,* syphili*s, Trichomonas vaginalis,* herpes simplex virus
Tattooing	Sporotrichosis, blood borne infections
Widow inheritance and similar rituals	HIV
Kumbha Mela (involving ritualistic bathing and dipping in rivers)	Diarrheal disease including cholera, respiratory infection including COVID-19
Mortuary rituals	Cholera, Ebola, kuru
Ritual side rolls	Cutaneous larva migrans
Circumcision in Jewish infants	Herpes simplex virus
Ablution (involving nasal irrigation)	*Naegleria fowleri* meningoencephalitis
Mass gatherings (e.g., Haj)	Various infections including meningococcal meningitis, cholera, influenza, viral hepatitis, COVID-19, and *tuberculosis*

HIV: human immunodeficiency virus; HTLV: human T cell leukemia virus.

## Data Availability

No data were used to support this study.
